# In vitro inhibition of *Escherichia coli* β-glucuronidase by aripiprazole

**DOI:** 10.1007/s43440-026-00869-z

**Published:** 2026-06-04

**Authors:** Kohsuke Sugiyama, Kensuke Sato, Hanako Aoki, Maho Kotori, Ryota Nakano, Noriko Hida, Maiko Kusano, Masahiro Akiyama

**Affiliations:** 1 Department of Legal Medicine, Graduate School of Medicine, Showa Medical University, 1-5-8 Hatanodai, Shinagawa-ku, Tokyo, 142-0064 Japan; 2 Department of Sports and Health Sciences, Graduate School of Biomedical Engineering, Tohoku University, 6-6-12, Aramaki Aza Aoba-ku, Sendai, Miyagi 980-8579 Japan; 3 Department of Adduct Biology and Gut Microbial Function, Clinical Research Institute for Clinical Pharmacology and Therapeutics, Showa Medical University, 6-11-11 Kitakarasuyama, Setagaya-ku, Tokyo, 157-8577 Japan; 4 Department of Clinical Research and Development, Graduate School of Pharmacy, Showa Medical University, Tokyo, Japan; 5 Department of Physiology, Graduate School of Pharmacy, Showa Medical University, Tokyo, Japan

**Keywords:** Gut microbiota, β-glucuronidase, Aripiprazole, Duloxetine hydrochloride

## Abstract

**Background:**

Gut microbial β-glucuronidase (GUS) regulates the deconjugation of glucuronidated compounds, thereby influencing the enterohepatic circulation of xenobiotics and endogenous metabolites. Although many orally administered drugs reach the intestinal lumen, their direct effects on microbial enzyme functions remain incompletely understood. In this study, we systematically examined the impact of ten commonly prescribed central nervous system (CNS)-active drugs on microbial GUS activity.

**Methods:**

GUS inhibition was evaluated using purified GUS from *Escherichia coli* (*E. coli*), *E. coli* cell lysates, and intact bacteria, with p-nitrophenyl β-D-glucuronide as the substrate. Intracellular drug accumulation was quantified by high-performance liquid chromatography-tandem mass spectrometry, bacterial growth was assessed by optical density, and GUS activity in mouse cecal contents was analyzed ex vivo. Molecular docking and molecular dynamics simulations were conducted to characterize drug–GUS interactions.

**Results:**

Purified GUS screening identified aripiprazole (ARI) and duloxetine hydrochloride (DLX) as inhibitors. In intact E. coli, ARI, but not DLX, suppressed intracellular GUS activity without affecting bacterial growth. ARI also accumulated in *E. coli* at higher levels than DLX. In ex vivo assays, ARI showed inhibitory effects on GUS activity. Computational analyses suggested that ARI and DLX may preferentially interact with distinct regions of GUS, with ARI exhibiting more favorable binding energetics.

**Conclusions:**

These findings suggest that specific CNS-active drugs may directly modulate gut microbial GUS activity in *E. coli* under experimental conditions. In particular, ARI inhibited intracellular GUS activity, raising the possibility that certain neuropsychiatric drugs may influence microbial metabolic functions in addition to their canonical pharmacological targets.

## Introduction

The gut microbiota, microorganisms that inhabit the lumen of the gastrointestinal tract, contribute to the maintenance of both intestinal and systemic homeostasis by enzymatically converting dietary and endogenous compounds into physiologically active compounds, and coexisting with the host [1, 2] Owing to its intimate relationship with the intestinal environment, the gut microbiota is highly susceptible to various chemical substances encountered in daily life, including heavy metals, pesticides, and cigarette smoke components, as well as therapeutic agents that enter the intestinal lumen. Orally administered prescription drugs and over-the-counter drugs represent a substantial and continuous source of chemical exposure that may directly or indirectly influence microbial function [3].

Previous studies have focused on chemical- or drug-induced alterations in the composition of the gut microbial communities [4–6]. However, little is known about how these exposures affect the metabolic activities of gut microorganisms, despite the fact that enzymatic functions are key determinants of microbial contributions to host physiology. Among microbial enzymes, β-glucuronidase (GUS) is one of the most extensively studied. GUS catalyzes the hydrolysis of glucuronide conjugates, releasing their parent aglycones that are reabsorbed through the intestinal wall [7]. This process has profound implications for the pharmacokinetics and toxicity of xenobiotics, including drugs such as the anticancer agent irinotecan, and for the enterohepatic circulation of endogenous compounds such as serotonin and estradiol [8, 9].

Recent advances in microbiome research have revealed that the gut microbiota influence the central nervous system (CNS) through a bidirectional communication network known as the gut–brain axis, which involves neural, endocrine, immune, and metabolic pathways [10]. This interaction regulates mood, cognition, and stress responses; and dysbiosis of the gut microbiota has been implicated in neuropsychiatric disorders, such as depression, anxiety, and autism spectrum disorder [11–13]. Given the central role of serotonin in both gastrointestinal and neural functions, as well as the involvement of GUS in enterohepatic circulation [14, 15], it is reasonable to hypothesize that alterations in GUS activity may modulate CNS physiology via the gut–brain axis. Thus, drugs that target the CNS may exert unrecognized effects not only through their direct pharmacological actions on host tissues, but also through their capacity to modulate microbial metabolic activity.

Despite this possibility, the effects of CNS-active drugs on key microbial enzymes, such as GUS, remain not fully known. To address this gap, we selected ten widely used CNS-active compounds, namely, methylphenidate (MPH), diazepam (DZP), dextromethorphan hydrobromide monohydrate (DXM), diphenhydramine hydrochloride (DPH), levetiracetam (LEV), caffeine (CAF), ibuprofen (IBU), acetaminophen (APAP), aripiprazole (ARI), and duloxetine hydrochloride (DLX), and systematically evaluated their effects on microbial GUS activity. By elucidating how these therapeutic agents influence gut microbial metabolism at the enzymatic level, this study aimed to provide new insights into drug–microbiota–host interactions and deepen the understanding of how CNS-active drugs may indirectly contribute to the modulation of host physiology through their actions on the gut microbiome.

## Material and methods

### Materials

ARI (A2496), DLX (D4223), LEV (L0234), DPH (D0423), and APAP (H0190), and p-nitrophenyl β-D-glucuronide (pNPG; N0618) were purchased from Tokyo Chemical Industry (Tokyo, Japan). DZP (045–18901), CAF (035–06795), IBU (098–02641) and DXM (041–21551) were obtained from FUJIFILM Wako Pure Chemical Corporation (Osaka, Japan). MPH was chemically synthesized and used in this study after structural confirmation using NMR spectroscopy. Phosphate buffer (PB; pH7.0; 37,242–55), phosphate-buffered saline (PBS; 14,249–24), protease inhibitors (25955–11), and purified GUS (UniProt ID: P05804; 16,944–02) were acquired from NACALAI TESQUE, INC (Kyoto, Japan). *Escherichia coli* K-12 (*E. coli*) were purchased from the National BioResource Project (NBRP, Shizuoka, Japan). All the other reagents and chemicals were of the highest grade available.

### Purified GUS inhibition assays by CNS-active drugs

The assays were performed using purified *E. coli* GUS, and the reagents were prepared in PB (pH 7.0). Reaction mixtures (200 µL total) contained 50 µL of purified GUS (0.002 U/µL), 50 µL of each CNS-active drug at the indicated concentrations, and 100 µL of pNPG (0.5 mM final concentration). After addition of the test compounds, mixtures were pre-incubated for 5 min at 37 °C. Reactions were initiated by the addition of pNPG and subsequently incubated at 37 °C for 2 h. Absorbance at 415 nm, corresponding to p-nitrophenol generated upon hydrolysis of pNPG by GUS, was measured every 30 min using SpectraMax® i3 or VERSAmax microplate readers controlled with SoftMax Pro software (Molecular Devices, San Jose, CA, USA).

### IC_50_ determination and mode of inhibition analysis

IC₅₀ values (half maximal inhibitory concentration) and the mode of inhibition of ARI and DLX on GUS were determined using purified GUS inhibition assays, as described in Section. “Purified GUS inhibition assays by CNS-active drugs”. For IC_50_ determination, ARI was tested at concentrations ranging from 10 to 125 µM, while DLX was tested at concentrations ranging from 10 to 500 µM to generate full inhibition curves. To determine the mode of inhibition, assays were performed using ARI and DLX at concentrations near their respective IC₅₀ values, while varying pNPG concentration from 25 µM to 10 mM (final concentration). Kinetic parameters were calculated, and the inhibition type was inferred using Lineweaver–Burk plots.

### GUS inhibition assay using *E. coli* lysates

*E. coli* cells were disrupted using a Beads Crusher µT-12 (TAITEC, Saitama, Japan) in PB (pH 7.0), and total protein was adjusted to 1 mg/mL. The resulting protein extract was divided into groups for subsequent use. Reaction mixtures (200 µL total) consisted of 50 µL of *E. coli* protein extract, 50 µL of ARI or DLX solution (50 µM final), and 100 µL of pNPG (4 mM final). Samples were pre-incubated with each drug for 5 min, followed by initiation of the reaction with pNPG and incubation at 37 °C for 2 h. Absorbance at 415 nm, corresponding to p-nitrophenol generated upon hydrolysis of pNPG by GUS, was measured every 30 min using the SpectraMax® i3 or VERSAmax microplate reader with SoftMax Pro software.

### Effect of drug exposure on GUS activity in intact *E. coli*

*E. coli* cells cultured in lysogeny broth (LB) medium were divided into control, ARI- and DLX-exposed groups; cells harvested by centrifugation at 2200 × g for 5 min were resuspended in PB (control), PB containing 100 µM ARI or 100 µM DLX, respectively. After incubation for 15 min at room temperature, cells were centrifuged, washed with PB, and resuspended in 500 µL PB. Protein extracts were then prepared using a Beads Crusher µT-12 (TAITEC) in PB (pH 7.0). This cell disruption process was conducted to minimize confounding effects from intracellular transport processes and to allow for a more direct assessment of enzyme–drug interactions. For the GUS activity assay, reaction mixtures (200 µL total) contained 100 µL of protein extract (0.5 mg/mL) and 100 µL of pNPG (4 mM final). Reactions were incubated at 37 °C for 2 h, and absorbance at 415 nm, corresponding to p-nitrophenol generated upon hydrolysis of pNPG by GUS, was measured every 30 min using the SpectraMax® i3 or VERSAmax microplate reader.

### Quantification of drug concentrations in *E. coli*

The drug concentrations in *E. coli* were quantified using a Nexera XR LC system coupled to an LCMS-8045 tandem mass spectrometer (Shimadzu, Kyoto, Japan). For sample preparation, 1 µL of DZP (2.5 mg/mL) was added as an internal standard (IS) to 100 µL of the supernatants obtained in Section. “Effect of drug exposure on GUS activity in intact E. coli”. Proteins were precipitated by adding 400 µL of acetonitrile, and the mixture was centrifuged. The resulting supernatant was diluted 1:100 with the initial mobile phase (mobile phase A: mobile phase B, 95:5).

a Kinetex XB-C18 column (2.1 × 100 mm, 2.6 µm) and a SecurityGuard Ultra C18 cartridge (Phenomenex, Torrance, CA, USA) was used for chromatographic separation. The column oven was maintained at 40 °C with a flow rate of 0.3 mL/min, and an injection volume was 5 µL. The mobile phase consisted of 10 mM ammonium formate with 0.1% formic acid in water (A), and 10 mM ammonium formate with 0.1% formic acid in methanol (B). The gradient program was as follows: 5% B at 0 min; 5–95% B from 0 to 7.5 min; 95% B from 7.5 to 10 min; and re-equilibration at 5% B from 10 to 15 min.

Mass spectrometric detection was performed using electrospray ionization in the positive mode. Source parameters were as follows: capillary voltage, ±4.5 kV; nebulizing gas flow, 3 L/min (N₂); heating gas and drying gas flow, 10 L/min (N₂); interface temperature, 300 °C; heat block temperature, 400 °C; and drying temperature, 526 °C. Full-scan spectra were acquired over a *m/z* range of 150–500. Quantification was performed using multiple reaction monitoring (MRM) with the following transitions: ARI: *m/z* 448.1 → 285.0 (quantifier, collision energy (CE) 26 eV); 448.1 → 98.1 (qualifier, CE 40 eV), DLX: *m/z* 298.1 → 44.0 (quantifier, CE 11 eV); 298.1 → 154.0 (qualifier, CE 6 eV), DZP (IS): *m/z* 285.1 → 154.1 (quantifier, CE 28 eV); 285.1 → 193.1 (qualifier, CE 29 eV).

### *E. coli* growth assay after ARI and DLX exposure

To confirm that the observed effects on GUS activity were not attributable to general cytotoxicity on *E. coli,* the growth of *E. coli* following drug exposure was evaluated. *E. coli* cultured in LB medium were collected by centrifugation at 2200 × g for 5 min, and the supernatant was discarded. The cells were divided into two groups: a control group (cells resuspended in 1.0 mL PB, pH 7.0), and a drug-exposed group (cells resuspended in 1.0 mL PB containing 100 µM ARI or DLX). After incubation at room temperature for 15 min, which is the same exposure period used in Section. “Effect of drug exposure on GUS activity in intact E. coli”, the cells were centrifuged again at 2200 × g for 5 min to remove the drug and resuspended in 1.0 mL of LB medium. A 10-µL aliquot of each suspension was inoculated into 990 µL LB medium and incubated at 37 °C for 16 h. Bacterial growth was monitored by measuring absorbance at 600 nm at multiple time points using SpectraMax® i3 or VERSAmax microplate readers controlled with SoftMax Pro software.

### Assessment of enzymatic activity in cecal contents after drug exposure

The cecal contents from wild-type C57BL/6J mice were suspended in PBS (10 mg/mL) and filtered to remove debris. The suspension was treated with dimethyl sulfoxide (DMSO; control) or ARI (final concentration: 100 µM) and incubated for 1 h at room temperature. After centrifugation, the pellets were resuspended in PB containing 1% protease inhibitor, homogenized, and centrifuged. Supernatants were concentrated using 3 kDa cutoff filters, and the protein concentration was adjusted to 0.05 mg/mL. For enzymatic assays, protein samples (0.05 mg/mL) were mixed with pNPG (final: 4 mM) and incubated at 37 °C. Absorbance at 415 nm, corresponding to p-nitrophenol generated upon hydrolysis of pNPG by GUS, was measured every 30 min using SpectraMax® i3 or VERSAmax microplate readers controlled with SoftMax Pro software.

### Docking analysis

The structure of GUS (PDB ID: 3K46) [16, 17] was obtained from the RCSB PROTEIN DATA BANK (RCSB.org) [18] as PDB file. The 3D structures of ARI and DLX were obtained from SMILES using the YASARA structure software [19]. Unnecessary water molecules of GUS for the docking simulation were removed, and the structure of GUS was divided into chains A and B, because the entire protein structure of GUS was too large for blind docking simulation. Subsequently, protein – ligand docking simulations were performed using AutoDock VINA according to the manufacturer’s instructions. The binding sites were screened based on the calculated binding energies and dissociation constants. MD simulations were performed using the top five candidate clusters according to the manufacturer’s instructions. The assumed contact receptor amino acid residues were visualized using UMAP. To compare the binding energies calculated by the MD simulations, the binding energies from 15 ns onwards were used. The results were visualized using Python (ver. 3.12.10), umap-learn (ver. 0.5.9), scikit-learn (ver. 1.8.0), matplotlib (ver. 3.10.3), Seaborn (ver. 0.13.2), pandas (ver. 2.3.1), numpy (ver. 2.3.1), plotly (ver. 6.2.0), upsetplot (ver. 0.9.0) and GraphPad Prism 10 (GraphPad Software, CA, USA).

### Statistical analysis

Statistical analyses were performed using the GraphPad Prism 10 software (GraphPad Software, San Diego, CA, USA). The results are presented as mean ± SEM or median with interquartile range. Equality of variances was assessed by F-test prior to conducting unpaired t-tests. Differences in drug concentrations after exposure and in enzyme activity in cecal contents were evaluated using Wilcoxon signed-rank test, as the same *E. coli* cultures in LB medium or cecal contents were divided into paired groups. Comparisons of GUS activity among three groups (control, ARI and DLX-exposed groups) were performed using Friedman test with Dunn’s multiple comparisons test. Comparisons of *E. coli* growth were performed calculating the area under the curve (AUC) from the growth curves by using the trapezoidal rule by R (ver.4.5.3) and using Wilcoxon signed-rank test. The differences in mean binding energies during the stable phase of the trajectory (15–20 ns) were evaluated using Kruskal-Wallis test followed by Dunn’s multiple comparisons test. Comparisons of binding energies between ARI and DLX with GUS were performed using unpaired t-test. Statistical significance was set at *p* < 0.05.

## Results

### ARI and DLX inhibit purified GUS enzyme

**Fig. 1:**
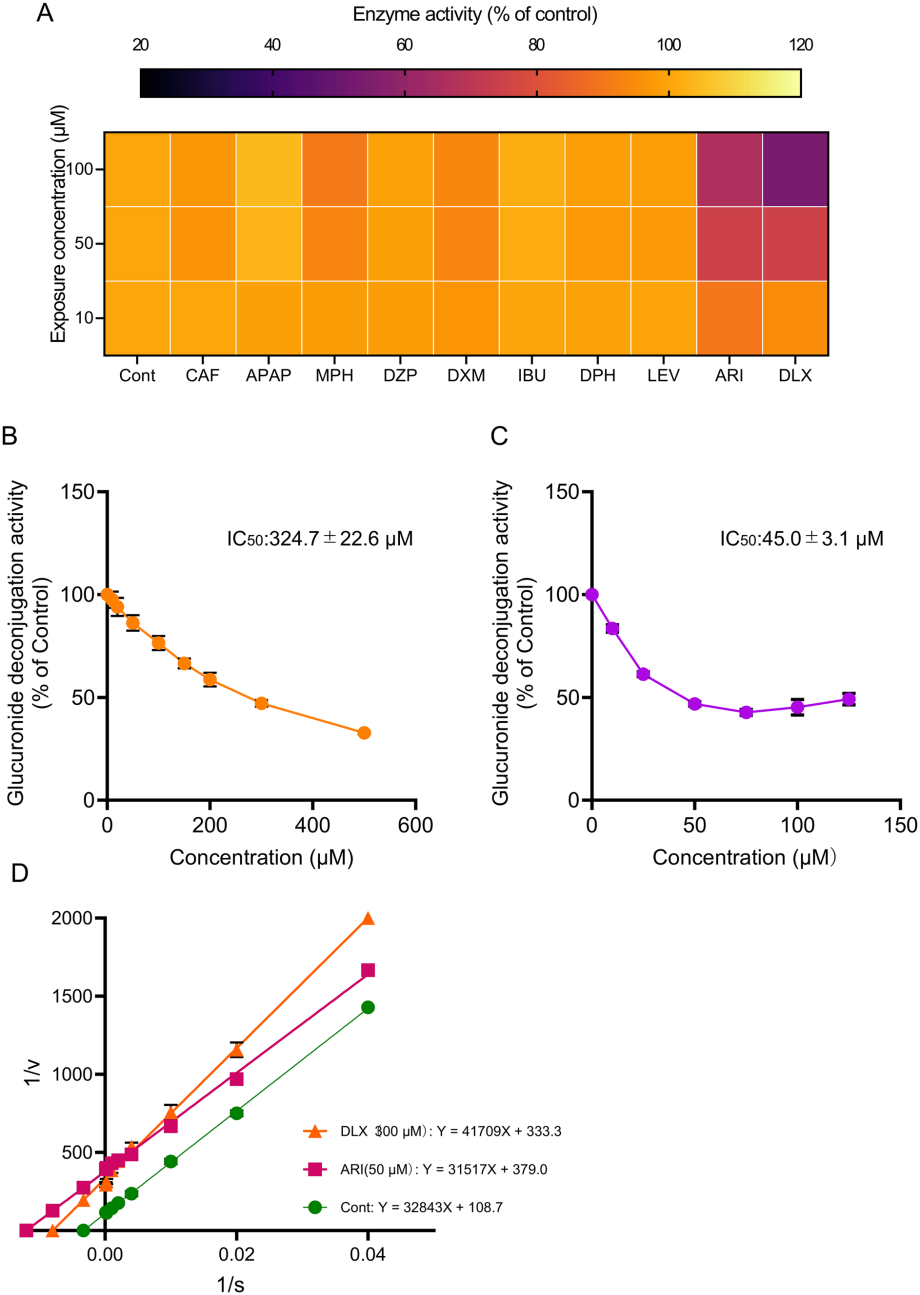
Screening of neuropsychiatric drugs for inhibition of purified GUS enzyme and IC_50_ evaluation of ARI and DLX. (**A**) Screening of ten neuropsychiatric or commonly co-administered compounds (CAF, APAP, MPH, DZP, DXM, IBU, DPH, LEV, ARI, and DLX) for inhibition of purified GUS activity. (**B**) Inhibition curve of DLX on GUS activity（IC₅₀ determination）using purified GUS. (**C**) Inhibition curve of ARI on GUS activity（IC₅₀ determination）using purified GUS. (**D**) Lineweaver-Burk plot in the presence of ARI or DLX at concentrations of near their respective IC_50_ values. The vertical axis represents the reciprocal of the reaction rate (1/v), and the horizontal axis represents the reciprocal of the substrate concentration (1/s). Purified β-glucuronidase (UniProt ID: P05804, 0.002 U/µL, 50 µL) was pre-incubated with each compound (50 µL) for 5 min at 37 °C. Subsequently, 100 µL of pNPG was added, and the reaction was allowed to proceed for 2 h at 37 °C. GUS activity was quantified by measuring the amount of p-nitrophenol produced. The concentration of pNPG was fixed at 1 mM (**A - C**) and ranged from 50 µM to 20 mM (**D**). ARI and DLX were used at concentrations of 50 µM and 300 µM, respectively, corresponding to approximately their IC₅₀ values. Data are presented as mean ± SEM from three independent biological replicates (*N* = 3). Abbreviations: APAP = acetaminophen, ARI = aripiprazole, CAF = caffeine, cont = control, DLX = duloxetine hydrochloride, DPH = diphenhydramine hydrochloride, DXM = dextromethorphan hydrobromide monohydrate, GUS = β-glucuronidase, IBU = ibuprofen, LEV = levetiracetam, MPH = methylphenidate, pNPG = p-nitrophenyl β-D-glucuronide, SEM = standard error of the mean

To examine the effects of structurally diverse neuropsychiatric drugs on GUS activity, purified GUS was incubated with ten clinically used compounds: CAF, APAP, MPH, DZP, DXM, IBU, DPH, LEV, ARI, and DLX. As shown in Fig. 1, ARI and DLX significantly reduced the GUS activity, whereas the remaining eight compounds showed no appreciable inhibitory effects. These data indicated that among the tested drugs, only ARI and DLX directly inhibited the purified enzyme. Based on these findings, the IC_50_ values of ARI and DLX, defined as the concentrations required to inhibit 50% of GUS activity, were determined using purified GUS: 324.7 µM ± 22.6 (SEM) for DLX (Fig. 1B) and 45 µM ± 3.1 (SEM) for ARI (Fig. 1C). Using concentrations near these IC₅₀ values, the mode of inhibition of ARI and DLX on GUS was further evaluated. Lineweaver–Burk analysis indicated that both Km (Michaelis constant; concentration of substrate at half -maximal enzyme reaction rate) and Vmax (maximal enzyme reaction rate) changed in the presence of ARI or DLX, suggesting that these compounds exhibit a mixed-type inhibition of GUS (Fig. 1D).

### ARI selectively inhibits GUS activity in *E. coli* without affecting bacterial growth

**Fig. 2:**
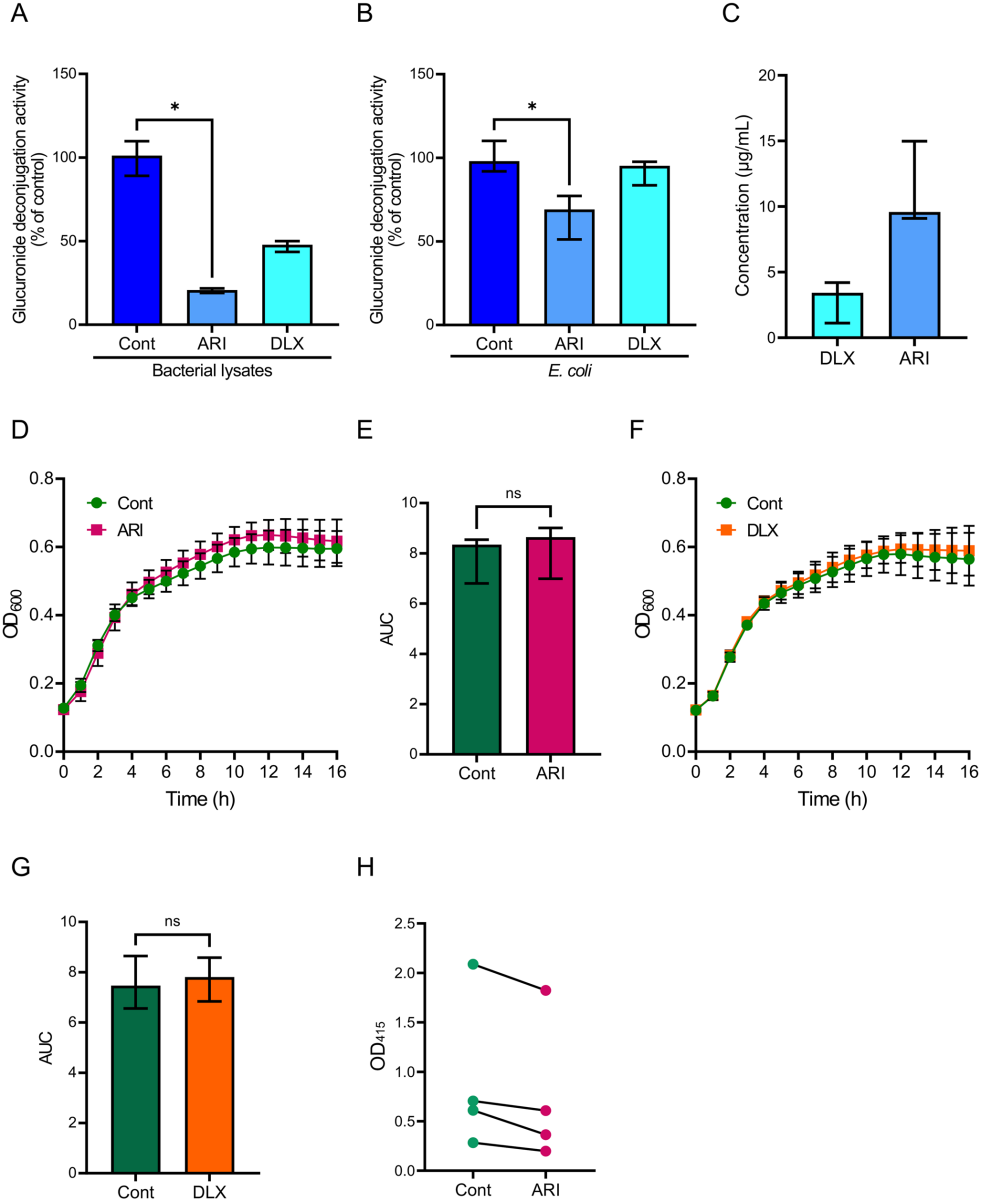
Effects of ARI and DLX on GUS activity in *E. coli* and mouse cecal contents. (**A**) GUS activity in *E. coli* lysates following incubation with 100 µM ARI or DLX for 2 h at 37℃. (**B**) Intracellular GUS activity in intact *E. coli* exposed to 100 µM ARI or DLX for 2 h at 37℃. (**C**) Intracellular concentrations of ARI and DLX quantified by HPLC–MS/MS. (**D**) Growth curves of *E. coli* following exposure or non-exposure to 100 µM ARI for 15 min. (**E**) AUC-based comparison of *E. coli* growth following exposure or non-exposure to 100 µM ARI for 15 min. (**F**) Growth curves of *E. coli* following exposure or non-exposure to 100 µM DLX for 15 min. (**G**) AUC-based comparison of *E. coli* growth following exposure or non-exposure to 100 µM DLX for 15 min. (**H**) GUS activity in mouse cecal contents following ex vivo exposure to 100 µM ARI for 2 h at 37℃. (**A, B, F**) GUS activity was quantified by measuring the amount of p-nitrophenol generated from pNPG. Concentration of pNPG was fixed at 8 mM. (**A, B**) data were analyzed by Friedman test with Dunn’s multiple comparisons test; (**C, D, E, F**) Wilcoxon signed-rank test. *p < 0.05, ns, not significant. Data are presented as mean ± SEM (**D, F**) or median with interquartile range (**A, B, C**), with *N* = 3 for all panels unless otherwise indicated. For panel H, *N* = 4 and individual data points are shown. Abbreviations: AUC = area under the curve, ARI = aripiprazole, DLX = duloxetine hydrochloride, *E. coli* = *Escherichia coli*, GUS = β-glucuronidase, HPLC–MS/MS = high-performance liquid chromatography–tandem mass spectrometry, pNPG = p-nitrophenyl β-D-glucuronide, SEM = standard error of the mean

Next, we assessed the effects of ARI and DLX in a monoculture of *E. coli,* a representative GUS-producing gut bacterium [20]. When *E. coli* lysates were incubated with each drug (100 µM) for 5 min prior to reaction with 50 µL of 8 mM pNPG for substrate, both ARI and DLX inhibited GUS activity as determined by measuring the absorbance of p-nitrophenol generated from pNPG, although only ARI showed a statistically significant difference. (Fig. 2A; Friedman test: x^2^ (2) = 6.000, *N* = 3, *p* = 0.0278; Dunn’s test: Control vs ARI; *p* = 0.0286, Control vs DLX; *p* = 0.4413), These results suggest that ARI retains inhibitory activity toward GUS in the presence of other cellular components. In contrast, when intact *E. coli* cells were exposed to the drugs (100 µM) for 15 min at room temperature, only ARI inhibited the intracellular GUS activity, whereas DLX had no effect (Fig. 2B; Friedman test: x^2^ (2) = 6.000, *N* = 3, *p* = 0.0278; Dunn’s test: Control vs ARI; *p* = 0.0286, Control vs DLX; *p* = 0.4413). To determine whether differences in intracellular accumulation accounted for this discrepancy, the intracellular drug concentrations were quantified by high-performance liquid chromatography-tandem mass spectrometry (HPLC–MS/MS). Although the difference did not reach statistical significance, ARI tended to accumulate in *E. coli* at higher levels than DLX (Fig. 2C; Wilcoxon signed-rank test, W = 6.000, *N* = 3, *p* = 0.25), suggesting that differential intracellular accumulation may contribute to the selective inhibition of intracellular GUS by ARI.

To evaluate whether GUS inhibition occurred independently of effects on bacterial viability, the growth of *E. coli* after exposure to the drug (100 µM ARI or DLX) was monitored by measuring absorbance at 600 nm during incubation at 37 °C (Fig. 2D and F). Growth was unaffected by ARI (Fig. 2E; Wilcoxon signed-rank test, W = 6.000, *N* = 3, *p* = 0.25), suggesting that ARI selectively inhibited GUS without impairing bacterial proliferation. Similarly, DLX had no significant effect on bacterial growth (Fig. 2G: Wilcoxon signed-rank test, W = 4.000, *N* = 3, *p* = 0.50)

Finally, to test whether ARI exerts similar effects in a more complex microbial community, GUS activity was measured in mouse cecal contents exposed to 100 µM ARI for one hour using a pNPG-based enzymatic assay. Although it cannot be ruled out that a portion of ARI was removed during protein extraction using a 3 kDa cutoff filter, ARI-treated samples showed a trend toward reduced GUS activity compared with controls. However, the magnitude of reduction varied among individual mice, and the overall difference did not reach statistical significance (Fig. 2H; Wilcoxon signed-rank test, W = −10.00, *N* = 4, *p* = 0.1250). These findings suggest that ARI may influence microbial GUS activity in a complex gut microbial environment, although further studies with larger sample sizes and in vivo validation will be required.

### Docking analysis suggests potential differences in ARI and DLX binding regions on GUS

**Fig. 3:**
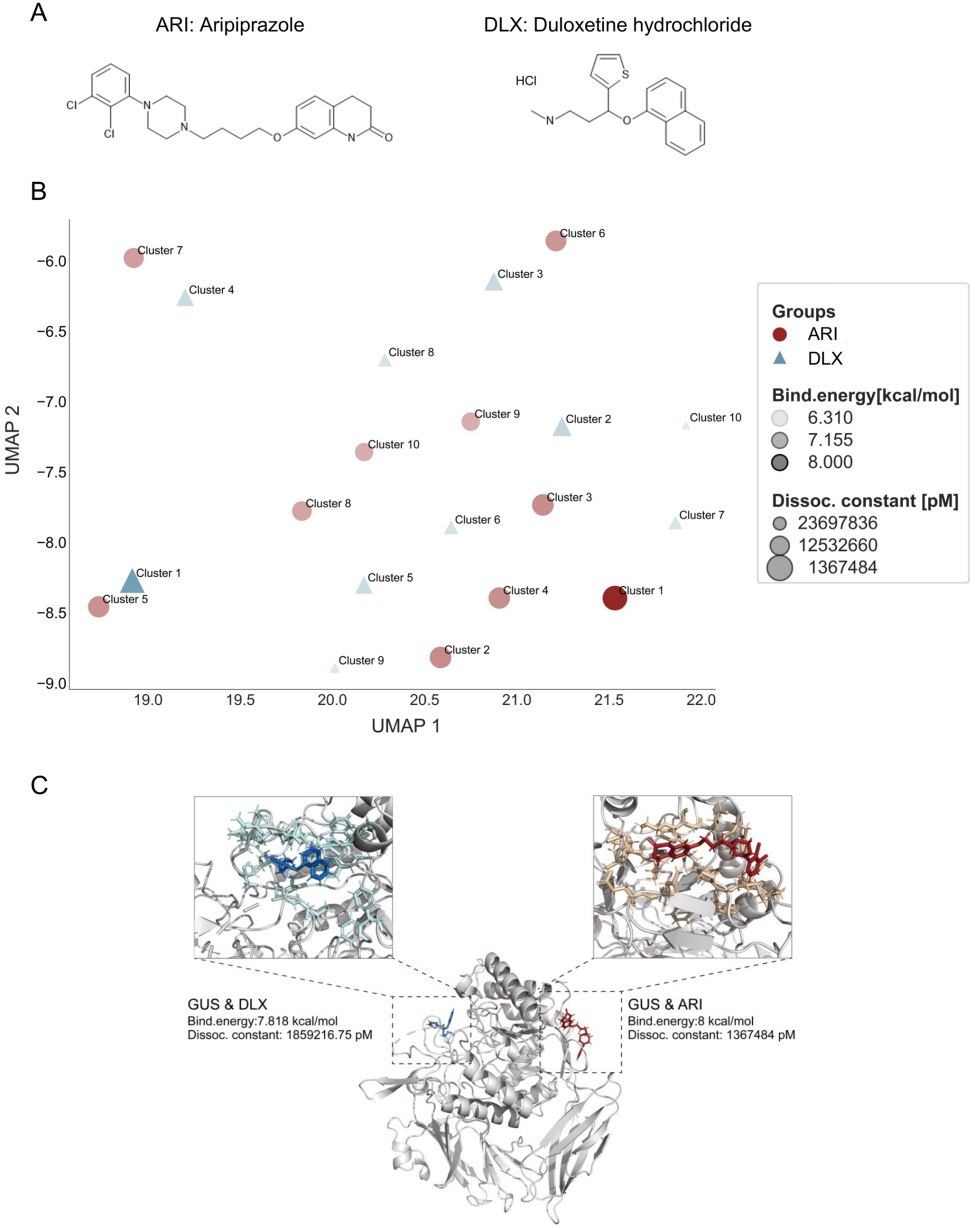
Blind docking simulation of GUS with ARI and DLX. (**A**) Chemical structures of ARI and DLX. (**B**) UMAP analysis of putative drug-enzyme binding sites obtained from blind docking analysis. Red circles indicate predicted ARI-binding residue clusters, and blue triangles indicate DLX-binding residue clusters. Darker colors correspond to higher predicted binding energies and larger symbols indicate lower dissociation constants. Cluster 1 exhibited the highest predicted binding affinity among the clusters. (**C**) Visualization of the GUS structure with the top-ranked docking poses of ARI and DLX. Red and blue indicate ARI or DLX, respectively. Beige and light blue represent the amino acid residue clusters involved in top ranked docking simulations (Cluster 1). Binding energy values are reported with inverted signs in YASARA; higher values indicate more stable binding affinity. Abbreviations: GUS = β-glucuronidase, ARI = aripiprazole, DLX = duloxetine hydrochloride

To explore the structural basis of GUS inhibition, blind docking simulations were conducted using the YASARA structure software [19]. Multiple putative binding energies, dissociation constants, and interacting with various receptor residues between GUS and ARI/DLX were predicted (Fig. 3A and B). It should be noted that YASARA reports binding energy values with inverted signs; thus, higher values indicate more stable binding.

To examine potential differences in ligand interaction patterns, the top 10 predicted contacting residue clusters for each ligand were analyzed using UMAP based on residue composition pattern (Tables 1 and 2, Fig. 3B). The resulting distribution showed that the top-ranked clusters (Cluster 1) of ARI and DLX were spatially separated in the UMAP space (Fig. 3C), suggesting differences in their interacting residue profiles. These results may indicate that ARI and DLX preferentially interact with distinct regions of GUS, although this interpretation remains speculative and requires experimental validation.

**Table 1:** Top 10 of blind docking simulation result between GUS and ARI

Cluster	Bind.energy [kcal/mol]	Dissoc. constant [pM]	Contacting receptor residues
1	7.818	1859217	ASP_163, LEU_361, GLY_362, PHE_448, TYR_469, TRP_471, TYR_472, THR_556, SER_557, GLY_559, ILE_560, LEU_561, ARG_562, ASN_566, LYS_568
2	6.72	11862458	HIS_107, GLN_108, GLY_109, GLY_110, THR_112, GLN_141, THR_142, ILE_143, AL, 353, GLU_383, GLN_386, AL, 387, GLN_390, AL, 391, GLU_394
3	6.656	13215613	TYR_160, PHE_161, HIS_162, ASP_163, LEU_361, GLY_362, LYS_370, TYR_469, THR_556, SER_557, GLN_558, GLY_559, ILE_560, LEU_561, ARG_562, ASN_566, LYS_568
4	6.61	14282550	GLU_279, GLN_280, PRO_287, PHE_288, TYR_289, SER_541, AL, 542, VAL_543, VAL_544, GLY_594, GLU_595, LYS_596, PRO_597, GLN_598
5	6.595	14648761	ASP_196, LEU_241, GLN_243, PRO_244, GLY_245, ILE_283, ASN_284, LYS_286, PRO_287, PHE_288, TYR_289, ASN_592, PHE_593, GLY_594, GLU_595
6	6.45	18710582	ASN_52, ARG_61, ASN_62, TYR_63, AL, 64, ASN_136, LYS_157, GLN_158, SER_159, TYR_160, PHE_165, ASN_166, THR_556, SER_557, GLN_558
7	6.431	19320328	PHE_161, HIS_162, ASP_163, LEU_361, GLY_362, PHE_448, TYR_469, TYR_472, THR_556, SER_557, GLN_558, GLY_559, ILE_560, LEU_561, ARG_562, ASN_566, LYS_568
8	6.431	19320328	TYR_160, HIS_162, ASP_163, LEU_361, GLY_362, TYR_469, TRP_471, TYR_472, THR_556, SER_557, GLY_559, ILE_560, LEU_561, ARG_562, ASN_566, LYS_568
9	6.339	22565834	HIS_107, GLN_108, GLY_109, GLN_141, THR_142, ILE_143, GLU_383, GLN_386, AL, 387, GLN_390, AL, 391, GLU_394
10	6.31	23697836	SER_-1, HIS_0, ASP_186, THR_188, TRP_204, VAL_206, LYS_393, ILE_396, AL, 397, LYS_400, LEU_435, ASP_436, PRO_437

**Table 2:** Top 10 of blind docking simulation result between GUS and duloxetine

Cluster	Bind.energy [kcal/mol]	Dissoc. constant [pM]	Contacting receptor residues
1	8.876	311749.469	GLN_17, PHE_154, GLU_210, VAL_213, VAL_224, PHE_226, ASN_277, TYR_341, PHE_370, PHE_387, TRP_389, TRP_452, GLU_459, TRP_460, ALA_461, GLN_462, PHE_468
2	8.493	595043.125	PHE_16, GLN_17, HIS_153, PHE_154, GLU_210, VAL_213, ALA_217, VAL_224, PHE_226, ASN_277, VAL_279, HIS_301, TYR_341, PHE_370, PHE_387, TRP_389, TRP_452, GLU_459, TRP_460
3	8.431	660685.938	SER_14, PHE_16, GLN_17, HIS_153, PHE_154, GLU_210, VAL_213, VAL_224, PHE_226, ASN_277, VAL_279, TYR_341, PHE_370, PHE_387, TRP_389, GLU_414, TRP_452, GLU_459, TRP_460, PHE_468
4	8.354	752379.25	SER_14, PHE_16, GLN_17, HIS_153, PHE_154, GLU_210, VAL_213, VAL_224, PHE_226, ASN_277, TYR_341, PHE_370, PHE_387, TRP_389, GLU_414, TRP_452, GLU_459, TRP_460, PHE_468
5	8.2	975709	GLN_17, HIS_153, PHE_154, GLU_210, VAL_213, VAL_224, PHE_226, ASN_277, VAL_279, HIS_301, TYR_341, PHE_370, PHE_387, TRP_389, TRP_452, GLU_459, TRP_460, ALA_461, GLN_462, PHE_468
6	8.131	1096220.63	GLN_17, PHE_154, GLU_210, VAL_213, GLN_221, VAL_224, PHE_226, ASN_277, VAL_279, HIS_301, TYR_341, ILE_344, PHE_370, PHE_387, TRP_389, TRP_452, GLU_459, TRP_460, GLN_462, PHE_468
7	8.098	1159010.25	GLN_17, PHE_154, GLU_210, VAL_213, ALA_217, VAL_224, PHE_226, TYR_341, PHE_387, TRP_389, GLU_414, GLU_459, TRP_460, ALA_461, GLN_462
8	8.087	1180729.5	PRO_4, LYS_6, PHE_7, PHE_8, LYS_146, PHE_148, TYR_204, ASP_265, SER_266, PRO_267, TYR_271, TRP_335, PRO_409, ILE_411, TYR_447
9	8.083	1188727.88	TRP_34, GLU_210, VAL_213, ALA_217, GLN_221, VAL_224, GLY_225, PHE_226, ASN_277, TYR_341, PHE_370, PHE_387, TRP_389, GLU_459, TRP_460, ALA_461, GLN_462
10	8.01	1344597	HIS_153, PHE_154, GLU_210, VAL_213, GLN_221, VAL_224, PHE_226, ASN_277, VAL_279, HIS_301, TYR_341, ILE_344, PHE_370, PHE_387, TRP_389, GLU_459, TRP_460, ALA_461, GLN_462

**Fig. 4:**
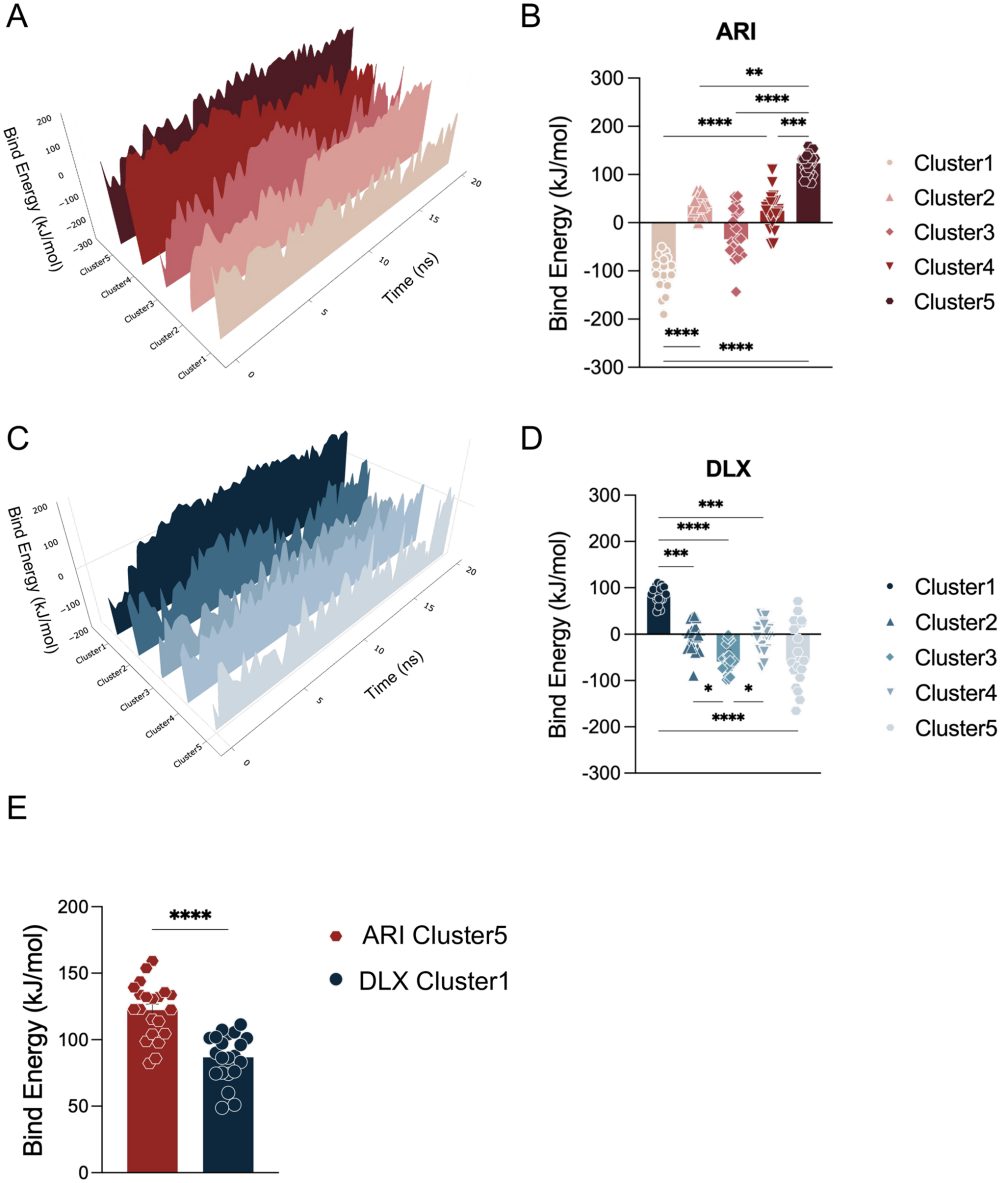
Molecular dynamics simulations of GUS binding with ARI or DLX. (**A, C**) Twenty-nanosecond MD simulations were performed for the top five docking clusters identified for each drug. (**B, D**) Binding energies of each cluster during the stable phase of the trajectory (15–20 ns) (*N* = 21 per cluster). For ARI, Cluster 5, ranked fifth in blind docking, showed the most stable binding in MD simulations. (**E**) Comparison of binding energies between ARI Cluster 5 and DLX Cluster 1. Overall, ARI exhibited higher binding energies than DLX (*N* = 21 per cluster). (**B, D**) Data are presented as median with interquartile range and were analyzed using Kruskal-Wallis test followed by Dunn’s multiple comparisons test. (**E**) Data are presented as mean and were analyzed using unpaired t-test. ** *p* < 0.01; *** *p* < 0.001; **** *p* < 0.0001. Abbreviations: MD = molecular dynamics, ARI = aripiprazole, DLX = duloxetine hydrochloride

To further evaluate the binding stability, molecular dynamics (MD) simulations were performed using the top five docking clusters for each compound (Fig. 4A, C). ARI exhibited the strongest affinity for Cluster 5 (Fig. 4B; Kruskal-Wallis: H = 83.50, *N* = 105, *p* < 0.0001; Dunn’s test: Cluster 5 vs Cluster 1 and 3; *p* < 0.01, Cluster 5 vs Cluster 2; *p* = 0.0089, Cluster 5 vs Cluster 4; *p* = 0.0003), whereas DLX showed the strongest affinity for Cluster 1 (Fig. 4D; Kruskal-Wallis: H = 62.29, *N* = 105, *p* < 0.0001; Dunn’s test: Cluster 1 vs Cluster 2; *p* = 0.0001, Cluster 1 vs Cluster 3 and 5; *p* < 0.0001, Cluster 1 vs Cluster 4; *p* = 0.0002). Comparison of the lowest energy states indicated that ARI exhibited a more favorable binding energy than DLX (Fig. 4E; unpaired t-test: t (40) = 5.901, *p* < 0.0001), consistent with the IC₅₀ values obtained under optimized conditions. This differs from the initial screening results, likely due to solubility limitations.

**Fig. 5:**
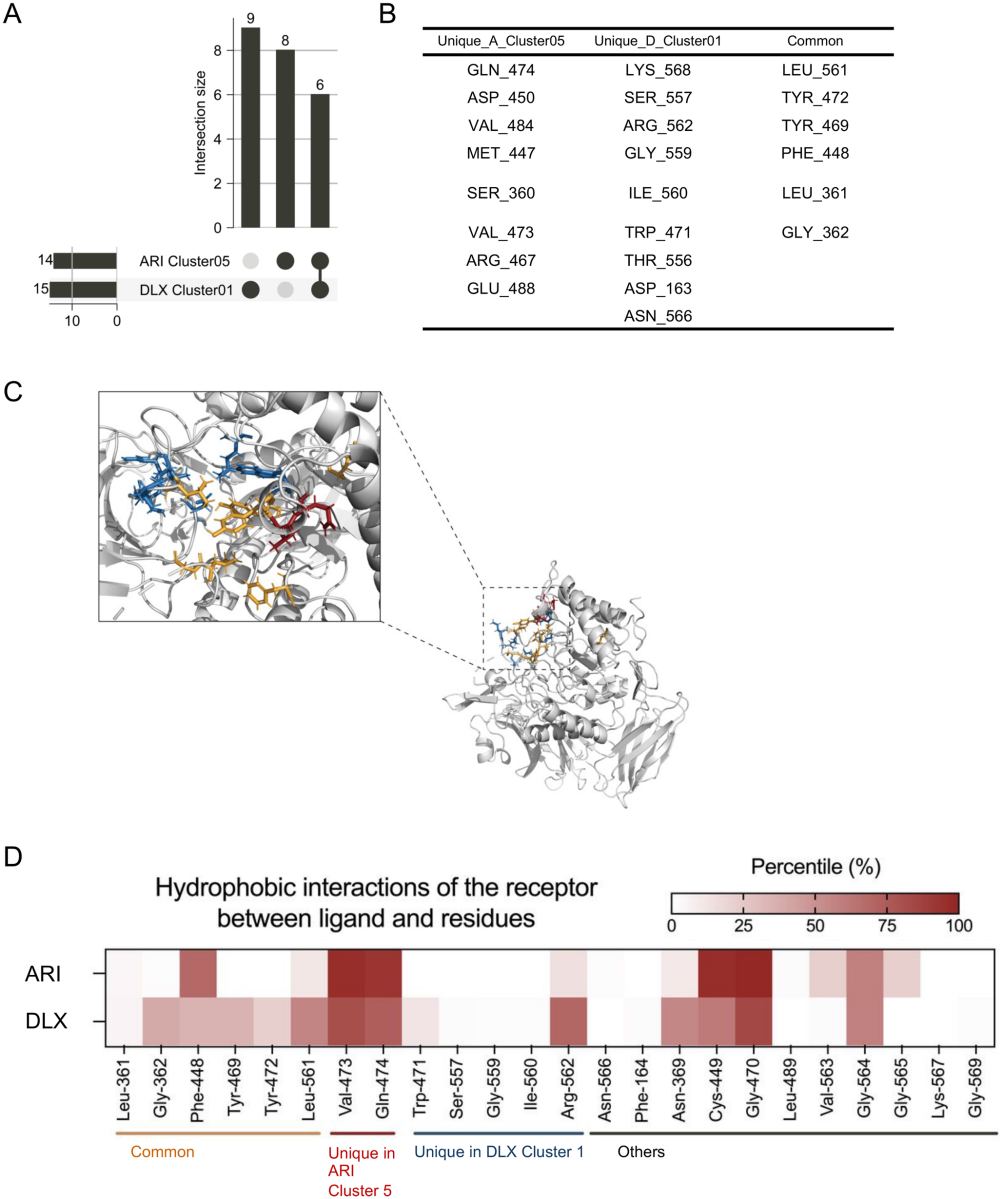
Amino acid composition of major binding clusters and hydrophobic interactions with ARI or DLX. (**A**) Intersection analysis of interacting residues between ARI Cluster 5 and DLX Cluster 1. (**B**) List of amino acid residues unique to ARI Cluster 5, unique to DLX Cluster 1, and common to both clusters. (**C**) Visualization of the GUS structure highlighting amino acid residues identified from the top-ranked MD simulations. Orange, red, and blue residues represent those unique to cluster 5 of ARI, unique to cluster 1 of DLX, and common to both ARI and DLX, respectively. (**D**) Heatmap of hydrophobic interactions between GUS and each drug, indicating that hydrophobic interactions are the predominant stabilizing forces. Abbreviations: ARI = aripiprazole, DLX = duloxetine hydrochloride, GUS = β-glucuronidase, MD = molecular dynamics

Further intersection analysis revealed that a substantial proportion of interacting residues differed between ARI Cluster 5 and DLX Cluster 1 (Fig. 5A and B). These residues were distributed in spatially distinct regions within the GUS structure (Fig. 5C), supporting the possibility of different interaction modes. In addition, MD simulations suggested that hydrophobic interactions are a major contributor to ligand binding (Fig. 5D).

Overall, these computational analyses provide a preliminary framework for understanding potential differences in ARI and DLX interactions with GUS, however experimental validation will be required to confirm the binding sites and underlying mechanisms.

## Discussion

This study demonstrated that ARI, but not DLX, inhibits intracellular GUS activity in *E. coli* without affecting bacterial growth. Because GUS is mainly localized in the cytoplasm and acts on glucuronide conjugates transported into the cell, intracellular drug exposure is essential for enzyme inhibition [21]. Consistent with this requirement, ARI accumulated in *E. coli* at markedly higher concentrations than DLX, suggesting why only ARI suppressed intracellular GUS activity. This difference in drug uptake is likely related to physicochemical properties; the logP value of ARI (4.55) is higher than that of DLX (~4.2), indicating greater lipophilicity and membrane permeability [22, 23].

Structural analyses further clarified potential drug–enzyme interactions. Blind docking and MD simulations suggested that both compounds may act through direct interference with GUS catalytic function. The interacting receptor residues identified for each drug were consistent with previously reported binding sites [16] or located in their vicinity. These regions comprise amino acid residues known to participate in substrate stabilization, suggesting that both compounds act through direct interference with GUS catalytic function [24]. ARI displayed a more favorable binding energy than DLX under the simulation conditions; however, these results should be interpreted with caution due to the limitations of computational modeling and differences from experimental assay conditions. Accordingly, the docking and MD analyses should be considered hypothesis-generating approaches that provide a framework for future studies.

Structurally, ARI contains a piperazine moiety, which is also present in other reported GUS inhibitors such as quetiapine [8], suggesting that cyclic amine structures may contribute to interactions with GUS. In contrast, DLX lacks this feature and appears to rely more on hydrophobic interactions, as indicated by MD simulations. These differences may contribute to variations in inhibitory behavior, although further experimental validation is required.

Overall, these computational analyses provide preliminary insights but do not establish definitive binding mechanisms.

Consistent with the in vitro findings, the analysis of mouse cecal contents showed that ARI also inhibited GUS activity in a mixed microbial environment; however, the magnitude of inhibition varied among individual mice. This variation likely reflects differences in the gut microbiota composition, suggesting that in humans, the extent of GUS inhibition may be personalized and contribute to individual variability in drug response.

Gut microbial GUS plays a central role in the deconjugation of glucuronidated metabolites, thereby influencing the enterohepatic circulation of endogenous molecules, such as serotonin, estradiol, and many xenobiotics [8, 9]. Our findings raise the possibility that ARI may modulate host physiology not only through its neuropsychiatric pharmacological actions but also by altering microbial enzyme function. Given that ARI has a long half-life (~60 h) and is excreted in the feces in high proportions (~60% of the administered dose) [25], prolonged exposure of gut microbes to the drug may induce an extended modulation of GUS activity. Such persistent changes could influence the metabolism of co-administered drugs or endogenous compounds, even after systemic clearance of ARI, thereby representing a potential new toxicity endpoint.

This study has some limitations. The sample size was relatively small, which may reduce statistical power and affect the precision of the estimates. To appropriately account for the data distribution, non-parametric statistical methods were applied; however, these methods may be less sensitive to detecting subtle differences between groups. Therefore, the results should be interpreted with appropriate caution.

The effects of the drugs on GUS reported here were evaluated using pNPG, an artificial substrate, rather than physiologically relevant endogenous glucuronide conjugates, such as serotonin glucuronide. Consequently, we did not directly assess the effect of ARI and DLX on GUS-mediated deconjugation of serotonin glucuronide or other endogenous glucuronides.

In addition, our analyses were restricted to in vitro experiments using *E. coli* and ex vivo assays using mouse cecal samples. We did not evaluate drug-induced alterations in gut microbiota composition or gene expression, nor did we consider polypharmacy, which is common among patients with schizophrenia or bipolar disorder [26–28].

Future studies should assess GUS activity, gut microbiota composition, and metabolic outcomes such as circulating levels of serotonin and other endogenous components in animals and humans receiving neuropsychiatric drugs. Such research will be essential to determine whether GUS inhibition occurs in vivo and to clarify how drug–microbe interactions contribute to inter-individual variability in therapeutic efficacy and adverse effects. Understanding how neuropsychiatric drugs modulate gut microbial enzymes may provide new insights into gut–brain axis mechanisms.

Moreover, the potential binding regions of ARI and DLX on GUS were inferred using molecular docking and MD simulations; however, these findings are predictive and require experimental validation. Future studies using approaches such as site-directed mutagenesis will be necessary to identify the binding sites.

Furthermore, while GUS activity was evaluated using mouse cecal contents as a complex microbial community, it should be noted that multiple GUS isoforms exist, including flavin mononucleotide (FMN)-binding GUS, which also contribute to the metabolism of endogenous substrates [8]. The effects of ARI and DLX on these different GUS types remain to be determined and warranted for further investigation.

In conclusion, this study suggests that prescription drugs may influence human physiology by modulating gut microbial enzyme activity independent of their primary pharmacological targets. Future investigations should systematically evaluate other commonly used drugs to determine how their interactions with the gut microbiota contribute to host metabolic regulation and drug responses.

## Data Availability

The datasets generated during and/or analyzed during the current study are not publicly available due to the data is saved in an unmodifiable format but are available from the corresponding author on reasonable request.

## Abbreviations

APAP: Acetaminophen
ARI: Aripiprazole
AUC: Area under the curve
BCA: Bicinchoninic acid
CAF: Caffeine
CE: Collision energy
CNS: Central nervous system
DLX: Duloxetine hydrochloride
DMSO: Dimethyl sulfoxide
DPH: Diphenhydramine hydrochloride
DXM: Dextromethorphan hydrobromide monohydrate
DZP: Diazepam
*E.**coli*: *Escherichia coli*
FMN: Flavin mononucleotide
GUS: β-glucuronidase
HPLC–MS/MS: High-performance liquid chromatography-tandem mass spectrometry
IBU: Ibuprofen
IS: Internal standard
LB: Lysogeny broth
LEV: Levetiracetam
MD: Molecular dynamics
MPH: Methylphenidate
MRM: Multiple reaction monitoring
PB: Phosphate buffer
PBS: Phosphate-buffered saline
pNPG: p-nitrophenyl β-D-glucuronide
SEM: Standard error of the mean
